# Therapy for BRAFi-Resistant Melanomas: Is WNT5A the Answer?

**DOI:** 10.3390/cancers7030868

**Published:** 2015-09-17

**Authors:** Chandra Prakash Prasad, Purusottam Mohapatra, Tommy Andersson

**Affiliations:** Cell and Experimental Pathology, Department of Translational Medicine, Lund University, Clinical Research Centre, Skåne University Hospital, Malmö SE-20502, Sweden; E-Mails: Chandra.Prasad@med.lu.se (C.P.P.); Purusottam.Mohapatra@med.lu.se (P.M.)

**Keywords:** melanoma, WNT5A, BRAFi, MAPK/ERK, PI3K-AKT, MITF

## Abstract

In recent years, scientists have advocated the use of targeted therapies in the form of drugs that modulate genes and proteins that are directly associated with cancer progression and metastasis. Malignant melanoma is a dreadful cancer type that has been associated with the rapid dissemination of primary tumors to multiple sites, including bone, brain, liver and lungs. The discovery that approximately 40%–50% of malignant melanomas contain a mutation in *BRAF* at codon 600 gave scientists a new approach to tackle this disease. However, clinical studies on patients have shown that although BRAFi (*BRAF* inhibitors) trigger early anti-tumor responses, the majority of patients later develop resistance to the therapy. Recent studies have shown that WNT5A plays a key role in enhancing the resistance of melanoma cells to BRAFi. The focus of the current review will be on melanoma development, signaling pathways important to acquired resistance to BRAFi, and why WNT5A inhibitors are attractive candidates to be included in combinatorial therapies for melanoma.

## 1. Introduction

Treatment options for malignant melanoma patients are limited, as metastatic melanoma easily becomes resistant to therapies (such as resistance to *BRAF* inhibitors, BRAFi). Significant advancements have also been achieved in the area of melanoma immunotherapy, with the development of drugs such as Ipilimumab (Human IgG1 antibody against CTLA-4) and Nivolumab (antibody against PD-1), but in the current review, the focus will be on *BRAF*-targeted therapies and the associated acquired resistance. We will also discuss the basis of melanoma disease, the signaling pathways associated with melanoma progression, and WNT5A signaling intervention as a new approach for future combinatorial therapy for malignant melanoma.

Light-skinned people are prone to melanoma, and the reason underlying this susceptibility is a genetic impairment. UV (ultraviolet) exposure increases the pigmentation of the skin, and this is mediated by the binding of α-MSH (α-melanocyte-stimulating hormone) to its cognate receptors, *i.e.*, MC1R (melanocortin 1 receptor). This ligand-receptor interaction triggers an intracellular signaling cascade that increases the production of melanin. Melanin synthesized by melanocytes relocates to keratinocytes and is responsible for absorbing and disintegrating UV energy [1]. Often, light-skinned people harbor germ-line polymorphisms in the MC1R gene that make them more susceptible to UV exposure [2,3,4]. Apart from inducing genetic changes in the skin, UV exposure is often linked to increase in the release of growth factors, production of reactive oxygen species and impairment of the cutaneous immune system and its functions [1,5]. Two other major risk factors that are associated with melanoma are family history and the presence of nevi (benign or atypical). To better understand these risk factors, one should comprehend the molecular changes associated with these factors and how they impact disease progression.

**Figure 1:**
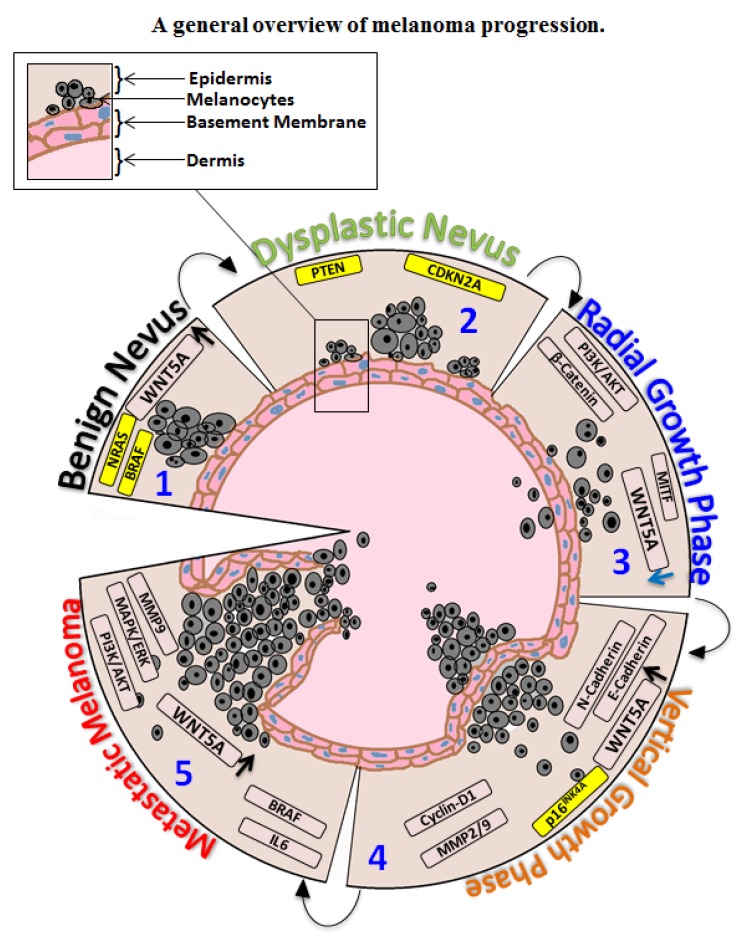
A general overview of melanoma progression. The schematic diagram is an adaptation of Clark’s different level model for melanoma development encompassing key molecules that are either mutated (marked in yellow) or exhibits an increased expression and that have been reported to play a role in *BRAF* inhibitor (BRAFi) resistance. The WNT5A molecule is unique as its expression is either increased or decreased during different phases of melanoma progression (indicated by the arrows). The numbers in the figure relates to the different phases of melanoma progression: Phase **1**, Benign Nevus; Phase **2**, Dysplastic Nevus; Phase **3**, Radial Growth Phase; Phase **4**, Vertical Growth Phase and Phase **5**, Metastatic Melanoma.

Cutaneous melanoma progression is a multi-step process and different staging systems have been established to clinically characterize disease progression, *i.e.*, Clark’s different level model [6], the Breslow’s thickness model [7] and TNM staging [8]. Clark’s model describes the series of histological changes associated with the progression of a melanoma from melanocyte to malignant melanoma [6] (Figure 1 illustrates a general overview of melanoma progression). The visibility of benign nevi (consisting of neval melanocytes) is the first phenotypic change; however, they rarely advance to cancer [6]. If one looks at the early mutations that occur at the benign nevi stage, mutations in the *BRAF* gene are crucial (approximately half of all melanomas harbor a mutation in *BRAF*). Active mutations in *N-RAS* have also been reported, and these are associated with 15% of melanomas. As both *N-RAS* and *BRAF* are upstream of mitogen-activated protein kinases-extracellular signal regulated kinases (MAPK-ERK) pathways, somatic mutations in these genes result in constitutive activation of ERK signaling, which is considered as integral pathway in melanoma development [9,10]. One reason why most nevi do not advance to cancer has been suggested to relate to the ability of mutant *BRAF* proteins to trigger senescence in human melanocytes by up-regulating INK4a (cell-cycle inhibitor of kinase 4a) expression [11]. However, the clinical findings that approximately 25% of melanomas arise from pre-existing nevi implicate that not all nevi undergo senescence [12,13] meaning that the previous finding [11] is not valid for all melanomas. Furthermore, in a more recent study, it was demonstrated that the current senescence markers are not able to distinguish nevi from melanomas thereby challenging the concept that nevi are growth arrested [14]. In addition to these problems, other effectors of nevi senescence have been suggested including; the p14-p53-p21 pathway, the FBXO31 pathway, the IGFBP7 pathway and the PI3K pathway [15]. Mutations in tumor suppressor genes (including *INK4*, *PTEN*) and changes in other signaling molecules can pave the way for melanoma development. The next step in Clark’s model is the formation of pre-malignant lesions or dysplastic nevus (Figure 1). Molecular changes thought to be associated with this phase are mutations in *CDKN2A* (cyclin-dependent kinase inhibitor 2A) and *PTEN* genes. Mutations in *CDKN2A* have been associated with 25%–40% of familial melanomas [16]. Loss of *CDKN2A* directly affects two tumor suppressor proteins, p16^INK4A^ and p14^ARF^ [17,18,19]. In melanomas, mutations in *PTEN* (phosphatase and tensin homologue), a tumor suppressor protein, have also been reported [20]. *PTEN* loss results in the accumulation of PIP_3_ (phosphatidylinositol (3,4,5)-trisphosphate) and activation of AKT proteins, which drives melanoma cell proliferation and survival [21]. Oncogenic mutations in *NRAS* and *BRAF*, coupled with loss of function mutations in *CDKN2A*, *p53* and *PTEN*, induce the melanoma radial growth phase (RGP). Increased levels of the phosphorylated (active) form of AKT protein are an indicatory factor of the RGP phase [22]. In Clark’s model, RGP is followed by the vertical-growth phase (VGP), which is characterized by the penetration of melanoma cells through the basement membrane (Figure 1). In VGP, melanoma cells loose cell-cell adhesion and undergo EMT (epithelial mesenchyme phenotype). Loss of E-cadherin and gain of *N*-cadherin has been reported during the melanoma transition from RGP to VGP [23,24]. Membranous loss of E-cadherin leads to the dissociation of β-catenin from the cell adhesion complex, which eventually translocates to the nucleus. An increased level of nuclear β-catenin positively regulates expression of CyclinD1 and MITF, which are associated with melanoma cell proliferation, survival and invasion [25,26].

## 2. Oncogenic Signaling in Melanoma

### 2.1. RAS/RAF/MEK/ERK Signaling

The RAS/RAF/MEK/ERK cascade plays an integral role in the pathogenesis of melanoma. It is regulated by cytokines, receptor tyrosine kinases and G-protein coupled receptors. Growth factors that constitutively activate this pathway are FGF (fibroblast growth factor), SCF (stem cell factor), HGF (hepatocyte growth factor) and GDNF (glial-cell-derived neurotrophic factor [27,28]. The activation of membrane receptors by these ligands activates the small G protein RAS, which further activates downstream effector RAF kinases (*ARAF*, *BRAF* and *CRAF*). RAFs then activate the kinases MEK1/2 and ERK1/2, which further phosphorylate nuclear and cytoplasmic substrates [29]. In humans, the most commonly mutated gene in this pathway is *BRAF*. *BRAF* has been shown to induce MAPK signaling, even without RAS activation. One of the important *BRAF* mutations involves exon 15, where valine is changed to glutamic acid at codon 600 (*BRAF^V600E^*), which induces a protein conformational change that makes the protein constitutively active [30,31]. The presence of *BRAF^V600E^* has been associated with poor survival in melanoma patients [32]. Inhibition of *BRAF^V600E^* results in a significant decrease in the metastatic potential of melanoma cells due to a reduction in cell extravasation [31]. In addition, the *BRAF^V600E^* mutation has also been positively correlated with pro-angiogenic growth factors such as VEGF, suggesting that a mutation in *BRAF* can be involved in the metastatic process [33]. *BRAF*-induced MAPK signaling further induces or activates ERK1/2 activity, thereby regulating cell proliferation, survival, migration and metastasis in melanomas.

### 2.2. The PI3K/AKT Pathway

PI3K (phosphatidylinositol 3-kinase)-AKT signaling is found to be frequently activated in melanomas, thereby controlling melanoma cell survival, proliferation and migration [34]. The PI3K-AKT pathway is activated by receptor tyrosine kinases (RTKs) or RAS. Activating mutations in *NRAS* (usually at codons Q60/61 and G12/13) are detected in approximately 20% of melanomas, which can result in activation of various pathways, including the RAS/RAF/MEK/ERK, protein kinase C (PKC), and PI3K/AKT pathways [35,36]. Mutations in the *c-KIT* receptor (which encodes RTK) are also responsible for activating the PI3K/AKT pathway, apart from the RAS/RAF/MEK/ERK and JAK/STAT pathways [37]. Mutations or amplifications in either *NRAS* or *KIT* can result in the activation of AKT, a downstream target of PI3K. Stronger staining of phospho-AKT (its active form) has been reported in metastatic melanoma samples compared to primary melanomas and nevus. Furthermore, similar to PI3K, high levels of active AKT have been associated with poor survival rates in melanoma patients [38,39].

Loss of function of *PTEN* is also crucial for the regulation of PI3K/AKT pathway. *PTEN* may be inactivated through missense mutations, frameshift mutations, focal or chromosomal deletions, epigenetic alterations or by microRNAs. Loss of *PTEN* was recorded in approximately 30% of cutaneous melanomas [21]. It has been demonstrated that the loss of *PTEN* in melanoma cells is associated with increased PI3K/AKT signaling [40,41]. Moreover, in melanomas, *PTEN* gene loss generally co-exists with the activation of *BRAF* mutations [42]. In an interesting study performed by Dankort *et al.*, the authors demonstrated that compared to *BRAF^V600E^* mice, which developed melanocyte hyperplasia, *BRAF^V600E^*/*PTEN^Null^* mice developed metastatic melanomas, suggesting that *PTEN* loss collaborates with the *BRAF^V600E^* mutation to induce metastasis [43]. Furthermore, proteomic analysis demonstrated that loss of *PTEN* protein in melanoma specimens and cell lines was positively associated with AKT activation, suggesting negative regulation of the PI3K/AKT signaling by *PTEN* [41].

### 2.3. WNT Signaling

Wnt signaling is an integral pathway in embryogenesis that is often found to be deregulated during carcinogenesis [44,45]. WNTs are a family of 19 secreted glycoproteins that can bind to different receptors or co-receptor complexes (including Frizzled (FZD), ROR1/2, RYK, and LRP), thereby triggering a range of cellular responses [46]. In their ability to transform mouse mammary cells, WNT ligands can be divided in three classes [47]; the highly transforming class that includes WNT1, WNT3A, WNT7A, WNT5B and WNT2; a non-transforming class, including WNT4, WNT5A and WNT6; and an intermediate transforming class, including WNT7B. However this grouping does not necessarily reflect which specific signaling that WNTs trigger. But, there are two main types of signaling pathways that can be induced by WNT ligands: canonical WNT/β-catenin signaling and non-canonical WNT signaling, including WNT/Ca^2+^ and the PCP (planar cell polarity) pathways. Of these we are going to address the former two pathways as they have been widely explored in the context of melanoma biology.

#### 2.3.1. WNT/β-Catenin Pathway

Unlike in most cancers, where β-catenin stabilization plays a positive role in cancer development, the role of β-catenin in melanoma is still controversial. Accumulation of β-catenin promotes metastasis in melanomas, but there are also reports that suggest that β-catenin overexpression suppresses invasion and is an indicator of a better prognosis in melanoma patients [48]. Compared to colon cancer, where mutations in *APC* and *CTNNB1* genes are responsible for constitutive WNT/β-catenin signaling, such mutations are rare in melanomas. However, activation of the WNT/β-catenin pathway was observed in approximately 30% of melanomas [49]. These data suggest that apart from β-catenin signaling, there are other factors that come into play during melanoma development. Experiments performed on transgenic mice revealed that melanocytes harboring constitutive expression of β-catenin were not adequate for melanocyte proliferation or for progression to melanoma [50]. In another study, activation of β-catenin increased metastasis in *NRAS*-driven melanomas, whereas mice carrying an *NRAS* mutation alone generated fewer tumors [25]. Studies on melanoma clinical samples have also suggested that patients harboring nuclear β-catenin have increased overall survival [51,52]. These findings are in parallel with the B16 murine melanoma model, where WNT3A expressing tumors, when implanted into mice led to decreased tumor proliferation and metastasis [53]. On contrary, there are also reports that suggest a direct role for β-catenin in melanoma metastasis [54,55]. Therefore, how β-catenin affects melanoma metastasis is still debatable, but there are certainly several other factors that might influence WNT/β-catenin signaling and its impact on melanoma.

##### MITF

MITF (Microphthalmia-associated transcription factor) belongs to the Myc superfamily of basic helix-loop-helix leucine zipper transcription factors [56] and is a downstream target of WNT/β-catenin signaling that has been reported to induce proliferative effects in melanocytes and melanoma cells [25,50]. In melanoma, the upregulation of MITF can regulate tissue-dependent regulation of CDK2 (cyclin-dependent kinase 2), which plays important role in melanoma proliferation [57]. Downregulation of MITF has been positively linked with melanoma cell migration and invasion [58]. It has been demonstrated that MITF-mediated downregulation of Dia1 (diaphanous-related protein) triggers actin re-organization, thereby leading to increased melanoma cell invasion [59]. However, melanoma patients harboring high levels of MITF have fewer metastases [60], suggesting that the regulation of MITF by diverse signaling pathways can have a distinct effect on melanoma cells. It has also been shown that *BRAF* triggers MITF expression in melanoma cells by inhibiting Brn-2, a transcription factor [61]. MITF can be regulated by various pathways; this might explain why the role of MITF in melanoma progression is still unclear.

#### 2.3.2. WNT5A Signaling

The initial indication that WNT5A is regulating cell migration came from the finding that WNT5A along with other non-canonical ligands (*i.e.*, WNT4 and WNT11) were implicated in regulating convergent extension (CE) in *Xenopus* embryo [62]. CE is development process driven by migration of polarized cells leading to tissue narrowing along one axis and elongation along perpendicular axis. During the gastrulation process, cells migrates into the embryo midline in order to undergo medio-lateral cell intercalation leading to anterior-posterior (A-P) extension [63]. Later it was shown that WNT5A had a similar effect on the polarized cell migration in *WNT5A*^−/−^ knockout mice, since these mice exhibited shortened A-P body axis [64]. These data was most likely the basis for the first investigation and demonstration that WNT5A positively regulates melanoma cell migration suggesting that it could promote metastasis [65]. WNT5A mRNA was found to be significantly higher in melanoma specimens compared to normal specimens [66]. In later studies, WNT5A mRNA expression was established as a robust marker of the metastatic melanoma phenotype [67]. Da Forno *et al.* demonstrated differential expression of WNT5A protein during melanoma progression, *i.e.*, high expression of WNT5A in nevi, decreased expression during RGP, followed by an increase in WNT5A expression in VGP and in metastatic lesions [68]. Other studies also implicate the increase of WNT5A mRNA in nevi [69,70] thereby suggesting that an increase in WNT5A expression might be one of factors that mediates decreased proliferation by inhibiting canonical WNT signaling at the nevi stage (Figure 1). However, subsequent downregulation in WNT5A expression during RGP allow upregulation of canonical WNT signaling with a decrease in E-cadherin expression and an increase in *N*-cadherin expression [24]. During VGP and metastasis, upregulation in WNT5A signaling through PKC has been shown to induce cell migration and EMT [65,71].

It has been demonstrated that highly proliferative melanoma cells express high β-catenin and low WNT5A, while more invasive melanomas express high WNT5A and low β-catenin [58]. Furthermore, WNT5A protein expression was positively correlated with melanoma progression, tumor grade and patient outcome [65,68]. WNT5A directs migration and invasion of melanoma cells in a PKC-dependent manner by inducing the expression of Slug, which induces EMT [71]. Moreover, the WNT5A-mediated effect on melanoma cell migration can be enhanced by the presence of syndecans 1 and 4, while WNT5A drives melanoma cell invasion and metastasis [46,72]. In addition, in a subset of melanoma cells, WNT5A has also been shown to induce β-catenin-mediated cell invasiveness via ARF-6 [73]. Apart from the established role played by WNT5A in melanoma migration and metastasis, its role as a regulator of melanoma phenotype switching has also emerged. WNT5A expression is inversely correlated with MITF in proliferative and invasive melanoma phenotypes [58,74]. WNT5A-mediated downregulation of LEF-1 (a transcriptional regulator of MITF) switches the melanoma cell phenotype from proliferative to invasive [75]. ROR1 and ROR2, receptors associated with WNT5A signaling, have also been implicated in WNT5A-induced phenotype switching, with ROR2 expressing melanoma cells demonstrating a more invasive phenotype [75].

We have demonstrated that the WNT5A antagonistic peptide Box-5 (a *t*-butyloxycarbonyl-modified WNT5A-derived hexapeptide) caused a significant inhibition of WNT5A-stimulated A2058 melanoma cell migration [76]. Furthermore, Box-5 also inhibited the basal migration in HTB63 cells by impairing WNT5A-induced PKC and Ca^2+^ signaling in these cells [76]. Proteomics screening revealed that WNT5A altered the expression of at least 174 different proteins in melanoma cell line and Box-5 inhibited the majority of these changes [77]. Pre-clinical studies suggest that some melanoma cell lines express very high amounts of the WNT5A protein. In such situations one need to use a very high concentration of the Box-5 peptide. To avoid such a high dose of Box-5, one might in parallel block the regulator(s) of WNT5A expression in melanoma. Recently, it was established that IL-6, a pro-inflammatory cytokine, can up-regulate the expression of WNT5A in melanoma cells, in a p38α-MAPK dependent manner [78]. In good agreement, the authors also demonstrated that Box-5 impaired IL-6-induced migration in melanoma cells [78]. These findings pave the way for a future combined therapy for simultaneous targeting IL-6 and WNT5A, resolving the problem of excessive usage of Box-5 peptide. Another aspect of Box-5 that deserves consideration is its half-life in blood. This is particularly important for a WNT5A antagonist but less so for a WNT5A agonist. In current pre-clinical trials differently modified Box-5 variants are being tested for their ability to impair melanoma cell migration and invasion, as well as for their half-life *in vivo*.

##### WNT5A Localization: A Topic of Debate

Researchers working on WNT5A biology understand a limitation of good antibody for the detection of WNT5A protein in clinical specimens. Several antibodies are commercially available for Western blot analysis and some of them can specifically detect WNT5A protein along with some non-specific bands. However, using the same antibody for immunohistochemical analysis on clinical samples can pose problems as they might detect non-specific staining. Many research groups have reported WNT5A staining in clinical specimens in several tumor types, including melanoma [51,68,74,77]. The general consensus is that WNT5A expression in melanoma is mainly cytoplasmic. However, along with the cytoplasmic staining some researchers reported WNT5A nuclear staining [51,68]. In one of these studies, the authors suggest that WNT5A nuclear staining might be an artifact related to the primary antibody used and/or the antigen retrieval condition employed [68]. This assumption is supported by the demonstration that nuclear WNT5A is not important in predicting survival in melanoma patients. To finally conclude if WNT5A nuclear staining is an artifact or not one need to identify the specificity of nuclear WNT5A staining including optimization of the immuno-histochemical staining protocol. It is possible that one also needs to analyze WNT5A expression in different cellular compartments (membranes, cytoplasm or nuclei) using cell fractionation.

## 3. *BRAF*-Targeted Therapy in Melanoma

In 2002, Davies H. *et al.* demonstrated the presence of oncogenic *BRAF* mutations (predominantly at codon 600) in approximately 70% of cutaneous melanomas, which revolutionized the treatment of advanced melanoma [9]. Later, a study performed by Long *et al.* demonstrated that the most common *BRAF* mutations in metastatic melanomas were *BRAF^V600E^* (approximately 74%) and *BRAF^V600K^* (approximately 20%) at Codon 600. As previously discussed, these active mutations in *BRAF* constitutively upregulate downstream MAPK/ERK signaling, thereby promoting cell proliferation and metastasis [79]. Two small *BRAF* inhibitors (BRAFi), Vemurafenib/PLX-4032 (Zelboraf^®^) and Dabrafenib (Tafinlar^®^), are now FDA-approved drugs that target mutant *BRAF* kinases (V600E and V600K). Two randomized Phase III trials that incorporated these inhibitors showed that patients using these drugs had significantly better survival rates compared to patients treated with Dacarbazine (an FDA-approved drug for melanoma) [80,81]. Similar effects were also observed in trials of small molecule inhibitors of MEK (e.g., Trametinib) in patients with *BRAF* mutations [80,82]. Because of the clinical success of BRAFi, there was a surge in efforts to identify patients with specific *BRAF* mutations by genetic screening. The Cobas^®^ 4800 *BRAF* mutation test (Roche Molecular Diagnostics, Pleasanton, CA, USA) is highly sensitive to V600E compared to V600K and V600D and was approved by the FDA to identify potential Vemurafenib patients. In this molecular test, DNA (standard 125 ng) is isolated from formalin-fixed or paraffin embedded melanoma tissue, and this procedure is followed by real-time polymerase chain reaction. Compared to Sanger sequencing, this test demonstrates fewer false negatives when detecting *BRAF^V600E^* [83]. A similar molecular test, the THxID *BRAF* Kit (identification of V600E and V600K), is also available for identifying potential Dabrafenib patients (FDA 2013). Unfortunately, the majority of the patients treated with BRAFi showed resistance to the drugs within 6–8 months of treatment [84,85]. Moreover, development of squamous cell carcinoma, which was present in nearly 20% of BRAFi treated patients, is also a concern [86].

### Signaling Pathways Responsible for Acquired Resistance to BRAFi

Mechanisms for resistance to BRAFi can be either intrinsic (pre-treatment factors) or acquired (developed during and post-treatment). Approximately 20% to 40% of melanoma patients do not respond to Vemurafenib (BRAFi) because of intrinsic resistance. In this part of the review, we will discuss the signaling pathways involved in acquired resistance to BRAFi.

MAPK signaling plays an important role in acquired resistance against BRAFi [84,85]. In a recently concluded Phase III study on 704 patients with metastatic melanoma (with *BRAF^V600^*), the use of a combination therapy of Dabrafenib (BRAFi) plus Trametinib (MEKi) led to improved overall survival, with no additional toxicity, compared to Vemurafenib (BRAFi) monotherapy [87]. However, another study demonstrated that patients with BRAFi-resistant melanomas up-regulated resistance mechanisms against combinatorial treatment (BRAFi + MEKi). These alterations were associated with similar genes that are involved in BRAFi resistance, thereby augmenting resistance to BRAFi + MEKi [88]. The genetic variations involved were gain of function of *BRAF^V600E^* and *NRAS^G12R^* (through amplification) and loss of function due to *PTEN^F127V^* deletion, which affected factors including *PTEN* and *CDKN2A*. MAPK reactivation has been further associated with mutations, where it results in a highly plastic RAF-MEK complex. The same study also confirmed clinical findings that showed acquired resistance to BRAFi + MEKi in melanoma cell lines [88]. As ERK is further downstream of RAS/RAF/MAPK/ERK signaling, there are studies that propose that ERK inhibitors (ERKi) might overcome BRAFi or BRAFi + MEKi resistance. In a different study, Carlino *et al.* recommended the use of ERKi over MEKi for the treatment of resistant melanoma cells. Moreover, they demonstrated that combining ERKi with PI3K/mTOR inhibitors promoted cell death in resistant melanoma cells [89]. Future molecular studies are required to determine the effects of ERKi and other therapeutic combinations on BRAFi or BRAFi + MEKi-resistant melanoma cells.

Apart from MAPK signaling, PI3K-AKT signaling is also involved in acquired resistance to BRAFi in melanoma. During BRAFi therapy, patients harboring normal *PTEN* genes exhibited genetic loss of *PTEN* [90]. In RTK overexpressing cells, mono-targeting PI3K-AKT had no effect on cell death; however, combinatorial inhibition of RAS/RAF/MAPK and PI3K-AKT resulted in significant cell death [91]. Shi *et al.* studied acquired resistance in melanoma tumors in patients treated with either Vemurafenib or Dabrafenib monotherapy. They demonstrated that, apart from MAPK signaling, the activation of PI3K-AKT is also important to acquired BRAFi resistance. They further showed that MAPK pathway inhibition can trigger increases in p-AKT levels [92]. Using genetically engineered mice, Marsh Durban *et al.* showed that silencing *PTEN* or PI3K activation through a mutation at *PIK3CA^H1047R^* is associated with *BRAF^V600E^* in melanoma metastasis [93]. In a separate study, the same group demonstrated that the combined blocking of PI3K and *BRAF^V600E^* significantly increased the durability of therapy responsiveness to an MEK1/2 inhibitor (GDC-0973) in *BRAF^V600E^*/*PIK3CA^H1047R^* or *BRAF^V600E^*/*PTEN^Null^* melanoma, suggesting that combined PI3K-mediated therapeutic intervention (with BRAFi) will be helpful in patients, whereas *PTEN* silencing and *PIK3CA^H1047R^* mutations are responsible for PI3K activation [94]. Moreover, AKTi (GSK2141795B) led to significant growth inhibition in various melanoma cell lines, including *PTEN*^−/−^ and AKT mutants. Long-term combinatorial treatment of AKTi with Dabrafenib and Trametinib (MAPK inhibitors) delayed drug resistance in a *PTEN*^−/−^ cell line [95].

The role of EGFR (epidermal growth factor receptor) signaling has also been implicated in BRAFi resistance. Approximately 8%–10% of colon cancers harbor *BRAF* mutations, which are associated with a poor prognosis. A study performed by Prahallad *et al.* demonstrated that inhibiting of *BRAF^V600E^* with Vemurafenib led to the activation of EGFR in a feedback manner, which supported proliferation in colon cancers. They further showed that expression of EGFR is sufficient to cause resistance to Vemurafenib in melanoma cells [96]. The EGFR/SFK/STAT3 signaling axis was found to be up-regulated in *BRAF*-resistant melanoma cell lines and in patients with intrinsic or who developed acquired resistance to Vemurafenib. However, combined treatment with an EGFR inhibitor and BRAFi was found to inhibit the growth of *BRAF*-resistant cells both *in vivo* and *in vitro* [97]. In another study, an inverse association was observed between MITF, Vemurafenib resistance and EGFR in tumor specimen and in melanoma cell lines. Moreover, the authors showed that forced expression of MITF reduced EGFR signaling in melanoma and colon cancer cells, which restored sensitivity to BRAFi/MEKi [98]. In Table 1 we have listed the most important factors that has been suggested to be responsible for acquired resistance to BRAFi.

## 4. WNT5A Signaling in BRAFi-Resistance and Therapeutic Interventions

In addition to MAPK-ERK, PI3K-AKT and EGFR signaling, WNT5A signaling has also been implicated in melanoma resistance to BRAFi therapy. Initial studies that suggested the role of WNT5A in acquired resistance to BRAFi (Vemurafenib/PLX4032) came from investigations performed by Tap *et al.* [99]. These investigators demonstrated that, of the six melanoma cell lines (carrying *BRAF* mutations) when treated with increasing doses of PLX4032, three cell lines (M288, SKEML28 and M14) attained resistance. Expression profiles of these three cell lines revealed that they expressed up-regulated factors including WNT5A, neuregulin, and endothelin 1 compared to their parental cells. All the three resistant cell lines also demonstrated the reactivation of MAPK signaling [99]. Another study that was performed on 11 *BRAF* mutant cell lines (either with low or high WNT5A expression) revealed a positive association between WNT5A expression and BRAFi resistance [100]. By stimulating cells with recombinant WNT5A (rWNT5A), the authors demonstrated that resistance to BRAFi (*i.e.*, PLX4720) increased two-fold in the BRAFi sensitive melanoma cell line 451 LU. Furthermore, by showing that knockdown of ROR2 can sensitize melanoma cells to PLX4720 therapy, they indicated a role for WNT5A in BRAFi resistance in melanoma cells. In parallel, they validated these findings *in vivo* by showing an increased response to PLX4720 therapy in ROR2 siRNA-treated PLX-resistant 1205 LU melanoma cells, compared to controls. Investigators extended their findings to clinical specimens as well when they demonstrated that 8 of 12 patients who relapsed while on BRAFi therapy showed increased positivity for WNT5A expression, clearly indicating the direct involvement of WNT5A in BRAFi resistance in melanoma patients. A similar trend was also reported in BRAFi-resistant melanoma tumors, where 7 of the 11 patients showed increased *WNT5A* expression compared to pretreated samples [101]. Investigators further demonstrated that BRAFi-resistant melanoma cell lines overexpressed WNT5A protein and that WNT5A expression was not affected by the removal of BRAFi. By using hierarchical clustering, investigators have shown that BRAFi-resistant cells harbor unique transcriptional program signatures that are associated with WNT5A expression, suggesting a clear role for WNT5A in BRAFi-mediated drug resistance [101].

One of the pathways that have been shown to be activated in BRAFi-resistant melanoma is PI3K-AKT, which can fuel the MAPK-ERK pathway, thereby driving proliferation and survival. We previously showed that WNT5A signaling can trigger the PI3K-AKT pathway in melanoma cells, thereby driving the aerobic glycolysis [77]. Furthermore, we demonstrated the ability of Box-5 (WNT5A antagonistic peptide developed in our laboratory) to inhibit extracellular lactate, suggesting that impairment of WNT5A signaling inhibits a glycolytic phenotype via inhibition of the PI3K-AKT pathway in melanoma cells. In parallel to our studies, exogenous treatment with WNT5A in UACC1273 and A2058 cell lines has been shown to increase the expression of phospho-AKT (ser473) [101]. Consequently, knockdown of WNT5A signaling via siRNA treatment in BRAFi-resistant A375 melanoma cells led to a significant decrease in phospho-AKT (active form) in a PI3K-dependent manner, suggesting that WNT5A regulates PI3K-AKT in melanoma [101]. Recently, Obenauf *et al.* analyzed the secretome of BRAFi resistant melanoma cells and they observed hyperactivation of several signaling pathways, in particular PI3K-AKT signaling [102]. Therefore, a treatment strategy that includes antagonizing WNT5A signaling (via Box-5) is likely to impair the PI3K-AKT pathway in BRAFi-resistant tumors.

As WNT5A is a prerequisite for migration and invasion in both normal and BRAFi-resistant melanoma cells, targeting WNT5A signaling provides a promising therapeutic option for melanoma disease management. Indirect inhibition of WNT5A signaling *in vitro* and *in vivo* with siRNAs of ROR2 and FZD7 has clearly demonstrated that impairment of WNT5A signaling can increase sensitivity to *BRAF* inhibitors [100,101]. Oncomed Pharmaceutical’s has developed an anti-FZD mAb named Vantictumab (OMP-18R5) as a means to inhibit melanoma progression. However, in addition to FZD7, Vantictumab recognizes and binds to four other FZD receptors (FZD 1, 2, 5 and 8). In human tumor xenografts, combined treatment of Vantictumab along with cytotoxic chemotherapy demonstrated tumor shrinkage and reduction in cancer stem cell numbers in various tumor types indicating reduced WNT/β-catenin signaling [103]. The drug is currently in Phase-I clinical trials for non-small cell lung cancer (NSCLC), pancreatic cancer and metastatic breast cancer. However, as indicated above one should be cautious using antibodies for melanoma treatment that targets FZD receptors involved in both non-canonical and canonical β-catenin signaling, since the latter has been associated with better prognosis in melanoma patients. In addition, there are other therapeutic alternatives to the anti-FZD7 mAb approach, including recombinant soluble FZD7 (sFzd7), small interfering peptides (RHPDs) to block FZD7 and small molecule inhibitor FJ9, inhibiting protein-protein interaction between FZD7-Dishevelled [104,105,106]. However, the question and concern remain and that is if anti-FZD7 therapy in melanoma effect β-catenin signaling. In conclusion, the ideal treatment alternative would be an antagonist that specifically impairs WNT5A signaling without blocking a simultaneous β-catenin signaling activity.

Because the expression of β-catenin has been demonstrated to sensitize BRAFi-resistant melanoma cells [107], and WNT5A signaling can negatively regulate WNT/β-catenin signaling in the BRAFi-resistant melanoma cells [101] one can forward a notion that Box-5 might indirectly affect the β-catenin expression levels thereby making BRAFi-resistant melanomas more sensitive towards BRAFi. However, direct studies are warranted to investigate whether the inhibition of WNT5A signaling via Box-5 increases β-catenin expression, thereby sensitizing BRAFi-resistant melanomas to BRAFi therapy. Inhibition of WNT5A signaling in melanoma will make them less invasive. However, it is tempting to speculate that inhibition of WNT5A signaling will also indirectly increase β-catenin signaling making these tumors more proliferative favoring a combined anti-WNT5A and anti-proliferative therapy. The ability of WNT5A to inhibit MITF, a downstream target of β-catenin, may be one cause of resistance in BRAFi-treated melanoma patients, as MITF depletion can activate EGFR signaling, which can confer BRAFi resistance [98]. WNT5A suppresses MITF and its downstream effector MART-1 (melanoma antigen recognized by T cells-1), which has also been positively correlated with melanoma cell migration [108,109]. We propose that interfering with WNT5A signaling will affects PI3K-AKT and the β-catenin-MITF-EGFR axis, which would make Box-5 therapy useful for not only BRAFi-resistant melanomas but also melanomas that do not carry *BRAF* mutations.

**Figure 2:**
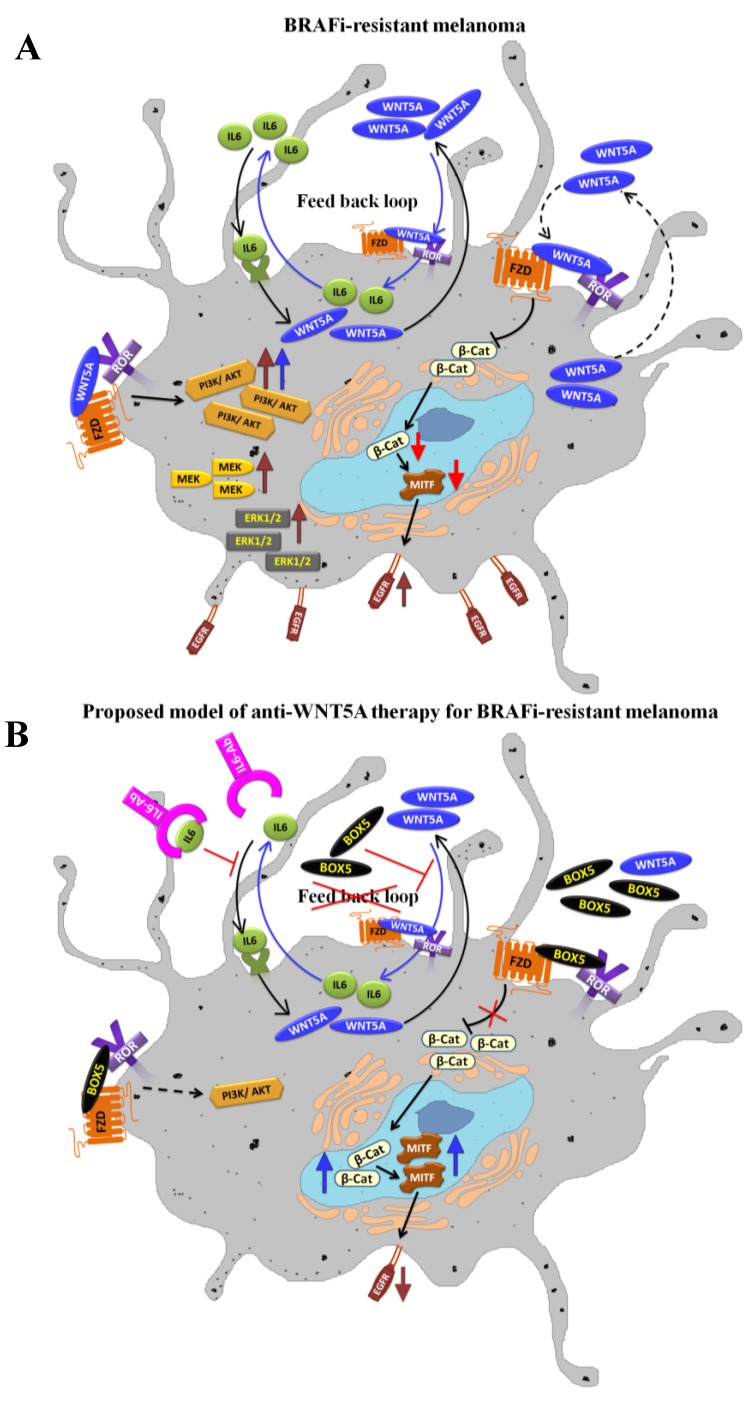
Proposed model of anti-WNT5A therapy in BRAFi-resistant melanomas. The schematic diagram summarizes the deregulated pathways involved in acquired BRAFi-resistance (**A**) and a hypothetical model for anti-WNT5A therapy (**B**).

We as well as others have demonstrated that WNT5A protein levels in different melanoma cell lines are variable and that sometimes the levels are very high. Such problems might be encountered in melanoma patients, too. To avoid excessive Box-5 usage, our laboratory investigated alternative means of targeting WNT5A signaling, such as identifying potential regulators of WNT5A expression in melanoma cells. We also investigated whether therapeutic interventions using such regulators, along with Box-5, can impair melanoma cells that express high levels of WNT5A. Our recent study on melanoma cells demonstrated that IL-6 can regulate expression levels of WNT5A in a p38α MAPK-dependent manner, thereby regulating melanoma cell migration [78]. In two *BRAF^V600E^* mutant cell lines (HTB63 and A375), we demonstrated that IL-6 can upregulate WNT5A protein expression, driving the migration of these cells. As WNT5A has been reported to be up-regulated in BRAFi-resistant tumors, there is also an increased possibility of enhanced IL-6 signaling in these tumors. On the basis of our previous findings we strongly propose a positive feedback loop whereby WNT5A regulates its own expression by indirectly regulating IL-6 in melanoma cells [78]. IL-6 itself plays an important role in melanoma progression. Studies based on transgenic mice demonstrated that mice lacking IL-6 develop fewer and smaller tumors, indicating a potential role for IL-6 in melanoma cell development [110]. Furthermore, Hoejberg *et al.* showed that melanoma patients with high IL-6 levels experienced shorter overall survival compared to patients with basal serum concentrations. In their study, they also found that patients with elevated levels of IL-6 and LDH had an increased risk of early death compared to patients with basal serum concentrations for both biomarkers [111]. Recently, Sos *et al.* demonstrated the possible role of autocrine IL-6 signaling in resistance to MAPK inhibition in a subset of *BRAF*-mutant melanoma cells [112]. Another important study performed on *BRAF^V600E^* mutant childhood astrocytomas showed that secreted IL-6 was involved in resistance to Selumetinib [113]. A schematic presentation of deregulated pathways involved in BRAFi-resistant melanoma cells and a hypothetical model for anti-WNT5A therapy that would contravene BRAFi-resistance is illustrated in Figure 2.

**Table 1:** Lists the factors suggested to be responsible for acquired resistance to BRAFi.

Factors	Signaling Involved	References
*BRAF^V600E^* copy number amplification /or alternative splicing	MAPK signaling	[92,114,115,116,117]
MEK1/2 activating mutations	MAPK signaling	[118,119,120,121]
*NRAS* activating mutations	MAPK signaling	[91,92,116,121,122]
*PTEN* loss	PI3K/AKT signaling	[40,121,123]
Mutations in PI3K/AKT pathway e.g., AKT1/3, PI3KCA *etc.*	PI3K/AKT signaling	[92,117]
MITF downregulation	EGFR signaling	[98,124]
WNT5A up-regulation	Non-canonical WNT signaling	[99,100,101]

Combined therapeutic approaches involving anti-IL-6 Ab (for antagonizing IL-6 signaling) and Box-5 (for antagonizing WNT5A signaling) might be beneficial for treating melanoma cells. Moreover, this combination might induce sensitivity to BRAFi because both biomarkers, either alone or in combination, are involved in BRAFi resistance in melanomas.

## 5. Future Perspectives

Given the current knowledge of deregulated signaling associated with BRAFi-resistant tumors, a combined anti-WNT5A therapy (Box-5 and anti-IL-6) provides an attractive therapeutic regime for the management of melanoma. WNT5A signaling has been shown to positively regulate melanoma cell invasion, and it is associated with reduced survival in melanoma patients. As recent studies have shown that WNT5A signaling is up-regulated in BRAFi-resistant tumors, impairment of WNT5A signaling will affect not only BRAFi-resistant melanomas but also melanomas that do not carry *BRAF* mutations. Future pre-clinical studies are warranted to explore the effect of anti-WNT5A therapies on BRAFi-resistant melanoma cells. These effects must be validated in WNT5A associated pathways, such as the PI3K-AKT, β-catenin-MITF-EGFR, and MAPK-ERK pathways. In addition, the consequences of anti-WNT5A therapies on the tumor microenvironment should also be investigated, as both IL-6 and WNT5A signaling can affect components of the tumor microenvironment, such as fibroblasts and immune cells.
